# Motivational interviewing and problem solving treatment to reduce type 2 diabetes and cardiovascular disease risk in real life: a randomized controlled trial

**DOI:** 10.1186/1479-5868-10-47

**Published:** 2013-04-19

**Authors:** Jeroen Lakerveld, Sandra D Bot, Mai J Chinapaw, Maurits W van Tulder, Piet J Kostense, Jacqueline M Dekker, Giel Nijpels

**Affiliations:** 1 Department of General Practice and the EMGO Institute for Health and Care Research, VU University Medical Center, van der Boechorststraat 7, Amsterdam, 1081 BT, The Netherlands; 2 Department of Epidemiology & Biostatistics and the EMGO Institute for Health and Care Research, VU University Medical Center, van der Boechorststraat 7, Amsterdam, 1081 BT, The Netherlands; 3 Department of Public and Occupational Health and the EMGO Institute for Health and Care Research, VU University Medical Center, van der Boechorststraat 7, Amsterdam, 1081 BT, The Netherlands; 4 Health Sciences and the EMGO Institute for Health and Care Research, VU University Medical Center, van der Boechorststraat 7, Amsterdam, 1081 BT, The Netherlands

**Keywords:** Type 2 diabetes, Cardiovascular disease, Prevention, Physical activity, Dietary behavior, Smoking, Lifestyle, Overweight

## Abstract

**Background:**

Intensive lifestyle interventions in well-controlled settings are effective in lowering the risk of chronic diseases such as type 2 diabetes (T2DM) and cardiovascular diseases (CVD), but there are still no effective lifestyle interventions for everyday practice. In the Hoorn Prevention Study we aimed to assess the effectiveness of a primary care based lifestyle intervention to reduce the estimated risk of developing T2DM and for CVD mortality, and to motivate changes in lifestyle behaviors.

**Methods:**

The Hoorn Prevention Study is a parallel group randomized controlled trial, implemented in the region of West-Friesland, the Netherlands. 622 adults with ≥10% estimated risk of T2DM and/or CVD mortality were randomly assigned and monitored over a period of 12 months. The intervention group (n=314) received a theory-based lifestyle intervention based on an innovative combination of *motivational interviewing* and *problem solving treatment*, provided by trained practice nurses in 12 general practices. The control group (n=308) received existing health brochures. Primary outcomes was the estimated diabetes risk according to the formula of the Atherosclerosis Risk In Communities (ARIC) Study, and the estimated risk for CVD mortality according to the Systematic COronary Risk Evaluation (SCORE) formula. Secondary outcomes included lifestyle behavior (diet, physical activity and smoking). The research assistants, the principal investigator and the general practitioners were blinded to group assignment. Linear and logistic regression analysis was applied to examine the between-group differences in each outcome measure, adjusted for baseline values.

**Results:**

536 (86.2%) of the 622 participants (age 43.5 years) completed the 6-month follow-up, and 502 (81.2%) completed the 12-month follow-up. The mean baseline T2DM risk was 18.9% (SD 8.2) and the mean CVD mortality risk was 3.8% (SD 3.0). The intervention group participated in a median of 2 sessions. Intention-to-treat analyses showed no significant differences in outcomes between the two groups at 6 or 12-months follow-up.

**Conclusions:**

The lifestyle intervention was not more effective than health brochures in reducing risk scores for T2DM and CVD or improving lifestyle behavior in an at-risk population.

**Trial registration:**

Current Controlled Trials: ISRCTN59358434

## Introduction

Overweight, smoking, low levels of physical activity and an unhealthy diet are major risk factors for chronic diseases such as type 2 diabetes (T2DM) and cardiovascular diseases (CVD) [1-3]. The high prevalence of these risk factors has become a major public health problem. More and more public health policy-makers expect health care providers to identify at-risk groups and to provide effective interventions to reverse this trend. It has been well established that intensive lifestyle interventions can lower the incidence of T2DM in individuals with impaired glucose metabolism. However, the question remains as to whether the positive results observed in these highly controlled settings can be achieved in primary health care settings [4,5]. A translation of efficacious interventions to ‘real life’ settings in order to evaluate its effectiveness is not a realistic option, because both the available resources and training facilities in primary health care would be exceeded [6]. Barriers in translating intensive interventions to a ‘real life’ setting include lengthy diagnostic testing procedures to identify pre-diabetes, the cost of highly educated personnel to provide the intervention, the possible cost of incentives to motivate participants, and offering the intervention in locations such as single medical centers [7].

So far, randomized controlled trials evaluating the effectiveness of programs that target lifestyle behavior(s) to prevent T2DM or CVD in primary health care settings have had various different results, and if they were effective, the effects were small and unsustainable [8-13]. Nevertheless, a number of non-randomized and small randomized studies have shown promising results [14-16]. To further inform the guidance of clinical practice and health care delivery there is a need for pragmatic trials in which high risk individuals are taught to change, independent of research staff. Such trials must be matching the targeted individuals' preferences, abilities and environmental constraints [17,18].

To address the current gap in the literature we developed and implemented a lifestyle intervention for the primary prevention of T2DM and CVD, tailored to the available resources and infrastructure for national primary health care services in the Netherlands.

The aim of this study was to investigate the effectiveness of a theory-based lifestyle intervention on the estimated risk of developing T2DM and CVD mortality in adults at risk, compared to providing written information only. A further aim was to assess the effects of the intervention on actual lifestyle behavior (physical activity, dietary behavior and smoking).

## Methods

### Study design and participants

**Figure 1:**
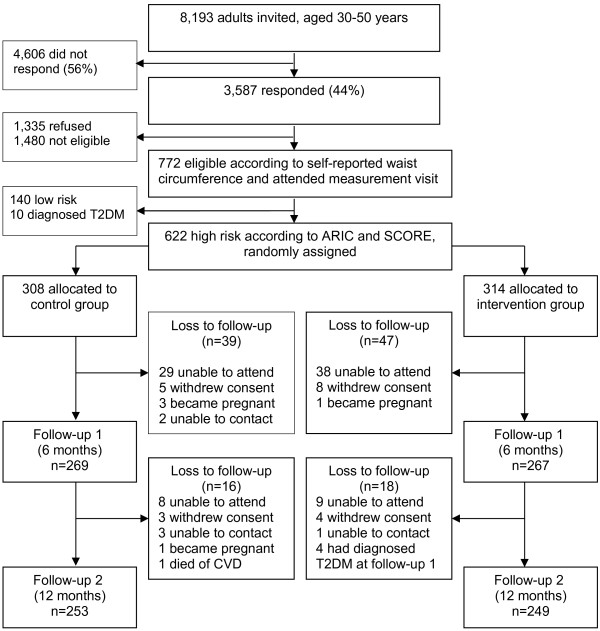
Flow chart of the Hoorn Prevention Study.

The methods and theory underlying the Hoorn Prevention Study, a parallel randomized controlled trial, have been reported in detail elsewhere [19]. Between December 2007 and April, 2008, a total of 8,193 men and women, 30–50 years of age, were sent an invitation letter asking them to participate in a selective screening procedure. The choice for the age group of 30–50 years was motivated by estimates of Dutch diabetes incidence rates based on the number of newly diagnosed patients by the general practitioner (GP) in five GP records [20]. Those estimates clearly mark the age-period in which incidence-rates start to rise (i.e. from 30 years onwards). The target group was approached after the identification of date of birth and absence of diabetes or known CVD from the computerized databases of the participating general practices (n=12). All invited individuals were living in various municipalities in the semi-rural region of West-Friesland in the Netherlands. The invitation included a tape measure for the measurement of waist circumference according to detailed instructions. Of the 3,587 respondents (43.8%), 2,401 responded positively, 921 of whom were eligible with regard to the pre-set cut-off score of the self-administered waist-circumference (≥101 cm for men and ≥87 cm for women). The cut-off scores were set 1 cm under the standard obesity cut-off point in order to account for a slight imprecision that may occur during self-measurement. Of these eligible respondents, 772 visited the Diabetes Research Center for baseline measurements, gave written informed consent, and participated in the trial (Figure 1).

T2DM and CVD risk scores were estimated according to the formulae described in the diabetes risk formula of the Atherosclerosis Risk In Communities (ARIC) Study [21] and the Systematic COronary Risk Evaluation (SCORE) project, respectively [22]. For both scores, and for each participant, age was extrapolated to 60 years to address the problem of a high relative but low absolute risk in younger persons. This made it possible to identify a potentially high risk at the age of 60 [22]. All respondents with at least a 10.0% T2DM risk and/or CVD mortality risk and no known prevalent T2DM or CVD were randomly assigned to either the intervention group or the control group. Before randomization, we excluded 150 individuals, 140 of whom had a less than 10.0% risk, and 10 who had undiagnosed T2DM (Figure 1).

The study protocol was approved by the Medical Ethics Committee of the VU University Medical Center in Amsterdam, and all participants gave written informed consent.

### Randomization

A randomization schedule was drawn up with a computerized random number generator. If more than one member of the same family participated, consecutive members of that family were randomized to the same group as the first member, to avoid contamination. To ensure concealment of the treatment allocation, an independent administrative assistant from the Diabetes Research Center, who had no information at all about the participants, performed the randomization. This was achieved by instructing the participants not to communicate about the intervention with their GP or the medical assistants.

### Intervention

The lifestyle intervention was based on the theory of planned behavior (TPB) [23] and the theory of self-regulation [24]. The intervention further built on the approach used in a study carried out by Welschen et al., who used similar intervention elements to change the lifestyle behavior of T2DM patients in order to decrease their CVD risk [25]. The intervention was provided by specifically trained practice nurses in the participating general practices. In six face-to-face 30-minute counseling sessions, followed by 3-monthly telephone sessions, *motivational interviewing*[26] (MI) and *problem solving treatment*[27] (PST) were used. The aim of MI was to strengthen the attitude and intention to change according to the TPB. An important method to achieve this was the focus on a discrepancy between the personal goals of the participants and their actual situation, as described in the theory of self-regulation. PST was used to support participants in finding solutions to overcome this discrepancy, to strengthen their perceived control, and to provide tools to overcome barriers that hinder changes in lifestyle behavior [27].

All practice nurses in the Hoorn Prevention Study received 18 hours of specific training from experienced psychologists prior to the intervention (12 hours of MI and 6 hours of PST). During the course of the study and the counseling sessions the practice nurses used a treatment manual developed by the project leader and the psychologists who provided the training. Practical coaching was provided halfway through the sessions, and consisted of one hour of individual coaching with feedback. A random selection of sessions per practice nurse, taped on a digital voice recorder, was used during the coaching. A peer supervision meeting was also arranged to provide ongoing feedback and to increase uniformity of the counseling style among the practice nurses.

### Control group

Participants in the control group received existing brochures containing health guidelines regarding physical activity and a healthy diet, obtained from the Dutch Heart Foundation. Smokers received an additional brochure on how to stop smoking from the Dutch Anti-Smoking Foundation (STIVORO). Control participants did not receive further contact or counseling.

### Outcome measures

The primary outcome measures were the estimated risk of developing T2DM and the estimated risk of CVD mortality. The 9-year risk of developing T2DM was estimated with the risk formula derived from data from the ARIC Study [21], based on ethnicity, parental history of diabetes, systolic blood pressure, waist circumference, and height. The 10-year risk of CVD mortality was estimated with the formula developed by the SCORE project [22], which includes sex, smoking status, total cholesterol, and systolic blood pressure.

Secondary outcome measures included:

•self-reported physical activity expressed in terms of i) metabolic equivalent of task (MET)- minutes per day light, moderate and vigorous activity (for classifying physical activity by rate of energy expenditure, i.e., by intensity) [28], and ii) number and proportion of participants who met the national recommendations for physical activity (≥30 minutes moderate-intensity physical activity such as brisk walking, on at least five days of the week) [29]. Physical activity was measured with a validated questionnaire [30].

•fruit intake (pieces per day, and number and proportion of participants who met the national recommendation of at least 2 pieces of fruit per day) and vegetable intake (grams per day, and number and proportion of participants who met the national recommendation of at least 200 grams of vegetable consumption per day). This was assessed according to an extended and modified version of the 8-item Food Frequency Questionnaire [31].

•smoking behavior (smoking every day/occasionally/never smoked), assessed with validated questions recommended by the World Health Organization (WHO) for the assessment of smoking status [32].

Height was measured to the nearest 0.1 cm without shoes using a wall-fixed stadiometer. Weight was measured to the nearest 0.5 kg, wearing light cloths and no shoes. The standard scales that were used (SECA; London, UK) were calibrated yearly. Waist circumference was measured midway between the lowest rib margin and the iliac crest. Two measurements to the nearest 0.5 cm were recorded; if the difference between the measurements was greater than 1 cm, a third measurement was made and the mean of the two closest measurements was calculated. Systolic and diastolic blood pressure were measured after 10 minutes of rest, in a seated position, with a Colin Press BP 8800p Non-Invasive Blood Pressure Monitor (Colin Medical Technology Corporation, USA). Fasting plasma glucose was measured according to the enzymatic reference method with hexokinase, HbA1c determination was based on the turbidimetric inhibition immunoassay for haemolysed whole blood, and total and HDL cholesterol and triglycerides were measured with the enzymatic colorimetric method. All laboratory tests were performed using the Cobas Integra system (Roche diagnostics, Basel, Switzerland). Data collection was done by medical assistants, who were unaware of the groups to which the participants were assigned.

### Sample size calculation

For the sample size calculation we used data from a Dutch working population of overweight people (body mass index ≥25), in which the standard deviation (SD) of the ARIC score was 8.11 [33]. For a two-sided detection of a 5.0% between-group difference in ARIC risk score (i.e. representing a difference in waist circumference of 1.5 cm or a difference in systolic blood pressure of 4 mmHg), with an alpha of 0.05 and a power of 90.0% in the present study, 120 participants per group were needed. However, in order to perform stratified analyses, and to take loss to follow-up into account, more participants were needed (i.e. 300 per group).

### Data-entry

In order to ensure a high level of data accuracy, 10.0% of the manually entered data was entered twice, and each second entry was checked against the first. A maximum discrepancy level of 1.5% was accepted.

### Statistical methods

Descriptive statistics (means (SD), or median and interquartile ranges, as appropriate) were used to describe the study sample with regard to demographics, physical characteristics and baseline laboratory values. Linear and logistic regression analysis was applied to examine the between-group differences in each outcome measure, adjusted for baseline values.

We examined effect modification by individual-level factors, including sex, age, level of education, and T2DM and CVD risk at baseline. Stratified analyses were performed for effect modifiers if their interaction term was considered to be significant (p<0.1 in this case). All primary analyses were performed according to the intention-to-treat principle: participants were analyzed in the groups to which they were originally randomly assigned, regardless of whether or not they actually received the intervention. Only participants for whom data were available were included in the analyses. In the analyses of smoking behavior we only included data of those who reported being a smoker at baseline. Participants with a fasting glucose >7.0 mmol/L (confirmed with a second fasting blood sample) were referred to their GP and then excluded from consecutive measurements because of the anticipated extra medical attention they might receive. Women who became pregnant during follow-up were also excluded because of potential bias in weight and waist circumference measurements. A per-protocol analysis included participants in the intervention group who attended at least 4 counseling sessions. All analyses were performed in SPSS 15.0 (SPSS Inc., Chicago, IL, USA).

## Results

Figure 1 presents the trial’s flow chart. 622 participants were randomly assigned to receive either the lifestyle intervention (n=314) or health brochures only (n=308). After 6 months, 536 participants (86.2%) attended the first follow-up measurement, 533 (85.7%) of whom provided complete data and could be included in the analysis. 502 participants (80.7%) attended the second follow-up measurement. A drop-out analysis showed no significant differences in baseline values of primary outcome measures between participants who completed the study and those who dropped out (T-test ARIC p=0.10 (95% CI −3.63 – 0.33); SCORE p=0.99 (95% CI −0.60 – 0.59)).

The baseline characteristics of the participants in the two groups were similar (Table 1). The mean age at baseline was 43.5 years (SD 5.3) and 363 participants were female (58.4%). Of 22 participants from the control group and 27 from the intervention group we also included the partner, who we assigned to the same group as the first-included. At baseline, the mean estimated 9-year risk of developing T2DM was 18.9% (SD 8.2) and the mean estimated 10-year CVD mortality risk was 3.8% (SD 3.0). Participants in the intervention group received a median of 2 (interquartile range 1–3) face-to-face counseling sessions and a median of 2.3 (interquartile range 1–3) sessions by phone. The baseline and follow-up values and the differences between groups in risk scores and lifestyle behavior are shown in Table 2.

**Table 1:** Baseline characteristics of randomized participants in the Hoorn Prevention Study

	**Control group (n=308) No. (%)**	**Intervention group (n=314) No. (%)**
**Sex**		
Female	185 (60.1)	178 (56.7)
**Age** (yrs), mean (SD)	43.4 (5.5)	43.6 (5.1)
**Level of education**		
≤Primary	103 (33.6)	101 (32.5)
Secondary	145 (47.1)	141 (44.9)
College, university	59 (19.2)	69 (22.0)
**Family history of diabetes**	77 (25.0)	94 (29.9)
**Anthropometrics**, mean (SD)		
Body weight (kg)	90.7 (15.4)	90.2 (15.5)
Waist circumference (cm)	96.7 (9.7)	96.7 (9.8)
**Blood pressure**		
Systolic (mmHg)	129.3 (13.3)	128.7 (13.2)
Diastolic (mmHg)	73.8 (9.0)	73.0 (9.9)

**Table 2:** Baseline and follow-up values and group differences in risk scores and lifestyle behavior (95% CI), adjusted for baseline

	**Control group**			**Intervention group**			**Group differences**	
	**Baseline**	**Follow-up 1 (6 months)**	**Follow-up 2 (12 months)**	**Baseline**	**Follow-up 1 (6 months)**	**Follow-up 2 (12 months)**	**β of between group difference Follow-up 1**	**β of between group difference Follow-up 2**
**Risk scores**								
ARIC	18.8 (8.5)	18.0 (7.6)	17.8 (9.2)	19.0 (7.8)	18.8 (8.5)	18.5 (8.3)	0.4 (−0.3 to 1.0)	0.3 (−0.6 to 1.2)
SCORE	3.8 (2.9)	3.7 (3.0)	3.7 (4.6)	4.0 (3.0)	4.0 (3.0)	4.0 (3.0)	0.0 (−0.3 to 0.2)	−0.2 (−0.7 to 0.4)
**Physical activity**								
light activities^a^	270 (150;371)	296 (150;399)	261 (137;364)	283 (163;392)	274 (171;393)	266 (171;378)	−13.3 (−36.6 to 10.1)	7.2 (−14.5 to 28.8)
moderate activities^a^	47 (19;120)	47 (19;121)	56 (26;126)	56 (19;150)	47 (21;120)	52 (21;138)	−9.5 (−22.3 to 3.2)	−9.4 (−22.0 to 3.2)
vigorous activities^a^	0 (0;17)	6 (0;17)	0 (0;17)	0 (0;17)	0 (0;17)	0 (0;17)	−0.8 (−3.3 to 1.8)	−0.1 (−3.3 to 3.1)
meeting recommendations n (%)^b^	184 (59.7)	167 (54.2)	160 (51.9)	201 (64.0)	161 (51.3)	162 (51.6)	OR 0.7 (0.5 to 1.1)	OR 0.9 (0.6 to 1.4)
**Dietary behaviors**								
pieces of fruit per day	1.1 (0.8)	1.3 (1.0)	1.2 (0.9)	1.1 (0.9)	1.1 (0.9)	1.1 (0.9)	−0.2 (−0.3 to 0.0)	−0.1 (−0.2 to 0.0)
meeting recommendations fruit intake n (%)^c^	67 (21.8)	70 (22.7)	68 (22.1)	63 (20.1)	57 (18.2)	58 (18.5)	OR 1.6 (0.9 to 2.6)	OR 1.4 (0.9 to 2.4)
vegetable intake (grams per day)	150 (70.4)	151 (68.5)	157 (89.9)	148 (69.5)	161 (126.6)	156 (74.6)	9.2 (−7.3 to 25.7)	−0.4 (−12.7 to 11.9)
meeting recommendations veg. intake n (%)^d^	63 (20.5)	57 (18.5)	56 (18.2)	72 (22.9)	55 (17.5)	62 (19.7)	OR 1.1 (0.7 to 1.7)	OR 0.9 (0.6 to 1.5)
**Smoking behavior**								
smokers n (%)	54 (17.6)	46 (17.2)	43 (17.0)	74 (23.9)	53 (20.0)	46 (18.3)	OR 0.5 (0.2 to 1.9)	OR 1.1 (0.4 to 3.1)

Data are mean (standard deviation) unless otherwise specified. CI= confidence interval.

ARIC= Atherosclerosis Risk In Communities formula based on: ethnicity (black yes/no), parental history of diabetes, systolic blood pressure, waist circumference and height.

SCORE= formula developed by the Systematic COronary Risk Evaluation project based on sex, smoking status (smoking yes/no), total cholesterol and systolic blood pressure.

^a^ Based on the Short QUestionnaire to ASsess Health enhancing physical activity (SQUASH). Values are median (Q1;Q3) *Metabolic Equivalent of Task* (MET)- minutes per day, representing the time engaged in specified physical activities multiplied by the metabolic equivalent value of each activity. Light activities are rated as 2·0 to < 4.0 METs, moderate activities are rated as ≥ 4.0 to < 6·5 METs, vigorous activities are rated as ≥ 6.5 METs.

^b^ Meeting national recommendations on physical activity (≥30 minutes moderate-intensity physical activity such as brisk walking, on at least five days of the week).

^c^ Meeting national recommendations on fruit intake (at least 2 pieces a day).

^d^ Meeting national recommendations on vegetable intake (at least 200 grams a day).

### T2DM and CVD risk scores

There were no significant between-group differences in either of the estimated risk scores between the intervention and the control group at either follow-up (Table 2).

### Lifestyle behavior

An increase in fruit intake of 0.2 pieces of fruit per day in the control group was found to be significantly different after 6 months, but not after 12 months (Table 2). We found no significant difference between the groups with regard to changes in physical activity, vegetable intake or smoking behavior over the 6 and 12 month follow-up period.

### Secondary analyses (per protocol, sub-group)

Per protocol analyses (n=360) did not affect the findings described above. Stratified analyses revealed that participants with a lower level of education in the control group were responsible for the increase in fruit intake. In this sub-group analysis the control group (n=308) consumed, on average, a fourth of a piece of fruit per day more than the intervention group (n=53) after 6 and 12 months. Stratified analyses of groups separated by the mean baseline ARIC or mean SCORE risk showed no change in the results.

## Discussion

In the current study we evaluated the effectiveness of an innovative, theory-based lifestyle intervention carried out in a primary health care setting. To our knowledge, we are the first to report on the effects of a lifestyle intervention to reduce the estimated risk of developing T2DM and CVD mortality. The cognitive behavior program was provided in the participants’ own general practice, by practice nurses instead of researchers in the study. At the same time, we were able to monitor the intervention carefully by providing standardized training for the nurse practitioners and feedback on the counseling sessions by means of tape recordings. In contrast to the procedure in former lifestyle interventions [4,5], the participants in our study were encouraged to find solutions instead of being told how to change their behavior, and they were also taught how to implement these solutions into their life. However, our findings show that the lifestyle intervention was not more effective than the provision of health brochures. Implementing lifestyle interventions in everyday practice poses challenging issues that require further investigation. A special focus in this regard may be the controllability of the dose of intervention in real-life settings, as this will probably be lower as compared to highly controlled settings. In addition, the role of participants’ social and physical environment may be of greater importance [34].

Earlier research in controlled settings has demonstrated that, separately, MI and PST are more effective than attention alone [35,36], and there is evidence to support the efficacy of MI in a number of programs promoting change in lifestyle behavior [36]. Although it has been convincingly demonstrated that T2DM can be delayed or prevented in high risk individuals, it is still a considerable challenge to provide evidence-based lifestyle programs for high risk populations in ‘real life’ settings. Few earlier randomized trials evaluated the effectiveness of diabetes prevention. These studies were, in line with our findings, not able to reproduce the very positive outcomes of previous efficacy trials [13,14]. Most diabetes prevention studies that were carried out in primary care had insufficient power, used single-group designs, and/or had high rates of attrition, and should therefore be interpreted with caution [37,38]. In the Hoorn Prevention Study we had sufficient power, randomization was performed at individual level, and relatively few participants were lost to follow-up. Other strengths include an appropriate design, choice of staff, staff training, adequate sample size and the choice for simple outcome measures. In addition, the participants did not receive financial incentives, and were recruited from the general population. Whereas the latter two arguments may be considered as strengths of the present study, they may also be associated with the lack of effectiveness of the intervention.

The findings of our process-evaluation indicate that the recruitment strategy was adequate and resulted in a reasonably high reach of the target population [39]. Practice nurses were competent and confident to provide MI and PST, and participant satisfaction was high. Nevertheless, the amount of attended sessions was low, and almost no effects on determinants of behavioral change were seen [39]. The rather low attendance rate may have contributed to the absence of an intervention effect, since a median number of 2 counseling sessions were attended. On the other hand, after per protocol analyses (which only included participants who had attended at least 4 counseling sessions) no change in the results were seen. Six or less face-to-face sessions as we provided may not have been enough to induce a sustainable lifestyle behavioral change, given that previous efficacious lifestyle interventions provided at least 12 sessions [40-42]. However, since the participants in our effectiveness study were not even motivated enough to attend 6 sessions, it is unlikely that they would be willing to attend more. It must also be mentioned that participants in our study were, on average, younger, and had a lower absolute risk of developing T2DM than those enrolled in previous effectiveness studies on lifestyle interventions. With regard to the external validity we like to point out that the study sample was not culturally diverse. Although our population (predominantly from a Western European culture) was representative for the study region in the Netherlands, this may affect the generalizability of findings.

Efforts were made to reduce barriers for participation to a minimum, as non-respondents are a potential threat to the external validity of the results [43]. We chose to approach potential participants via correspondence at multiple moments, as described by Dillman et al. [44]. Other efforts to reduce non-participation included the choice to provide the intervention in general practices (which are near to the homes of the participants), as well as to minimize the burden of the measurements by using short questionnaires, and to refrain from using unpleasant measurement methods such as 2-hour oral glucose tolerance tests.

After 6 and 12 months the lifestyle intervention was not more effective than the provision of health brochures in improving estimated risk scores for T2DM and CVD or lifestyle behavior in an at-risk population. Hence we conclude that the provision of this primary prevention approach was not effective in a Dutch ‘real life’ primary care setting.

## Abbreviations

ARIC: Atherosclerosis risk in communities; CVD: Cardiovascular disease; MI: Motivational interviewing; PST: Problem solving treatment; SCORE: Systematic coronary risk evaluation; T2DM: Type 2 diabetes.

## Competing interests

The authors declare that they have no competing interests.

## Authors’ contributions

JL, SDMB, MJMC, MWvT, JMD and GN were involved in the conception and design of the study. Initial data analyses and interpretation were done by JL and PJK. JL wrote the initial draft of the paper, and all authors were involved in critically revising the manuscript for important intellectual content. All authors read and approved the final manuscript.
